# Dissociation between asymmetric value updating and perseverance in human reinforcement learning

**DOI:** 10.1038/s41598-020-80593-7

**Published:** 2021-02-11

**Authors:** Michiyo Sugawara, Kentaro Katahira

**Affiliations:** 1 Department of Cognitive and Psychological Sciences, Nagoya University Nagoya, Aichi, Japan; 2 Research Fellowship for Young Scientists of Japan Society for the Promotion of Science, Tokyo, Japan

**Keywords:** Cognitive neuroscience, Decision

## Abstract

The learning rate is a key parameter in reinforcement learning that determines the extent to which novel information (outcome) is incorporated in guiding subsequent actions. Numerous studies have reported that the magnitude of the learning rate in human reinforcement learning is biased depending on the sign of the reward prediction error. However, this asymmetry can be observed as a statistical bias if the fitted model ignores the choice autocorrelation (perseverance), which is independent of the outcomes. Therefore, to investigate the genuine process underlying human choice behavior using empirical data, one should dissociate asymmetry in learning and perseverance from choice behavior. The present study addresses this issue by using a Hybrid model incorporating asymmetric learning rates and perseverance. First, by conducting simulations, we demonstrate that the Hybrid model can identify the true underlying process. Second, using the Hybrid model, we show that empirical data collected from a web-based experiment are governed by perseverance rather than asymmetric learning. Finally, we apply the Hybrid model to two open datasets in which asymmetric learning was reported. As a result, the asymmetric learning rate was validated in one dataset but not another.

## Introduction

Reinforcement learning (RL) models have been broadly used to model the choice behavior of humans and other animals^1,2^. Standard RL models suppose that agents learn action-outcome associations from outcomes on a trial-and-error basis^3^. The learned action values are assumed to be updated according to the reward prediction error, which is the difference between the actual and expected rewards^4,5^.

Although this mechanism is often assumed to underlie many background processes of human behavior, human decision making is subject to many biases^6^. Several modeling studies investigating human choice behavior have reported that the magnitude of the value update is biased depending on the sign of the reward prediction error. This bias can be represented in RL models as asymmetric learning rates for positive and negative outcomes^7–9^.

Lefebvre et al.^10^ suggested that this learning asymmetry reflects positivity bias (the tendency to emphasize good outcomes) in factual learning in which feedback is given only for the option chosen by the subject. Refining this idea, Palminteri et al.^11^ reported that this learning asymmetry represents confirmation bias (the tendency to selectively process information that supports one's beliefs) in counterfactual learning in which feedback is given for both the chosen and unchosen options^12,13^. These learning asymmetries lead to choice repetition because the influences of the outcomes that reinforce the choice (positive outcome for the chosen option and negative outcome for unchosen option) are enhanced, whereas those that weaken the choice are diminished^14^.

It has also been shown that our decisions depend on our choice history regardless of the choice outcome^15–18^. A positive dependency leads to the repetition of the same choices (hereafter, “perseverance”). Perseverance leads to behavior seemingly similar to that resulting from asymmetric learning rates. Katahira^14^ suggested the possibility that the estimation of asymmetric learning rates suffers from statistical artifacts caused by model misspecification. If an RL model without the perseverance factor is fitted to data that possess intrinsic autocorrelation (e.g., perseverance), the model tends to represent perseveration by asymmetric learning rates. Thus, a statistical bias that overestimates the difference in learning rates occurs. Due to this statistical bias, it is difficult to identify the cognitive process underlying human choice behavior. Nevertheless, the identification of computational processes, such as asymmetric value updating and perseverance, is crucial for interpreting neural mechanisms and investigating the association with personality traits in the fields of neuroscience, psychology, and psychiatry^7,8,10,19–23^.

The present study proposes methods to dissociate these computational processes from empirical behavioral data. Specifically, we address this issue by using a Hybrid model incorporating asymmetric learning rates and perseverance (hereafter Hybrid model). In the present study, we first conduct simulations to investigate how the Hybrid model works to identify the true underlying processes under various conditions. Then, we demonstrate how the Hybrid model can identify the underlying process in an empirical dataset with a relatively large sample size. Finally, to clarify the genuine process underlying open datasets collected from previous studies reporting asymmetric updating, we apply the Hybrid model to these datasets. According to a series of investigations, we conclude that the Hybrid model combining outcome-based and outcome-independent processes enables the detection of the genuine cognitive process underlying choice behavior while avoiding statistical artifacts.

## Results

### Instrumental learning paradigm

**Figure 1:**
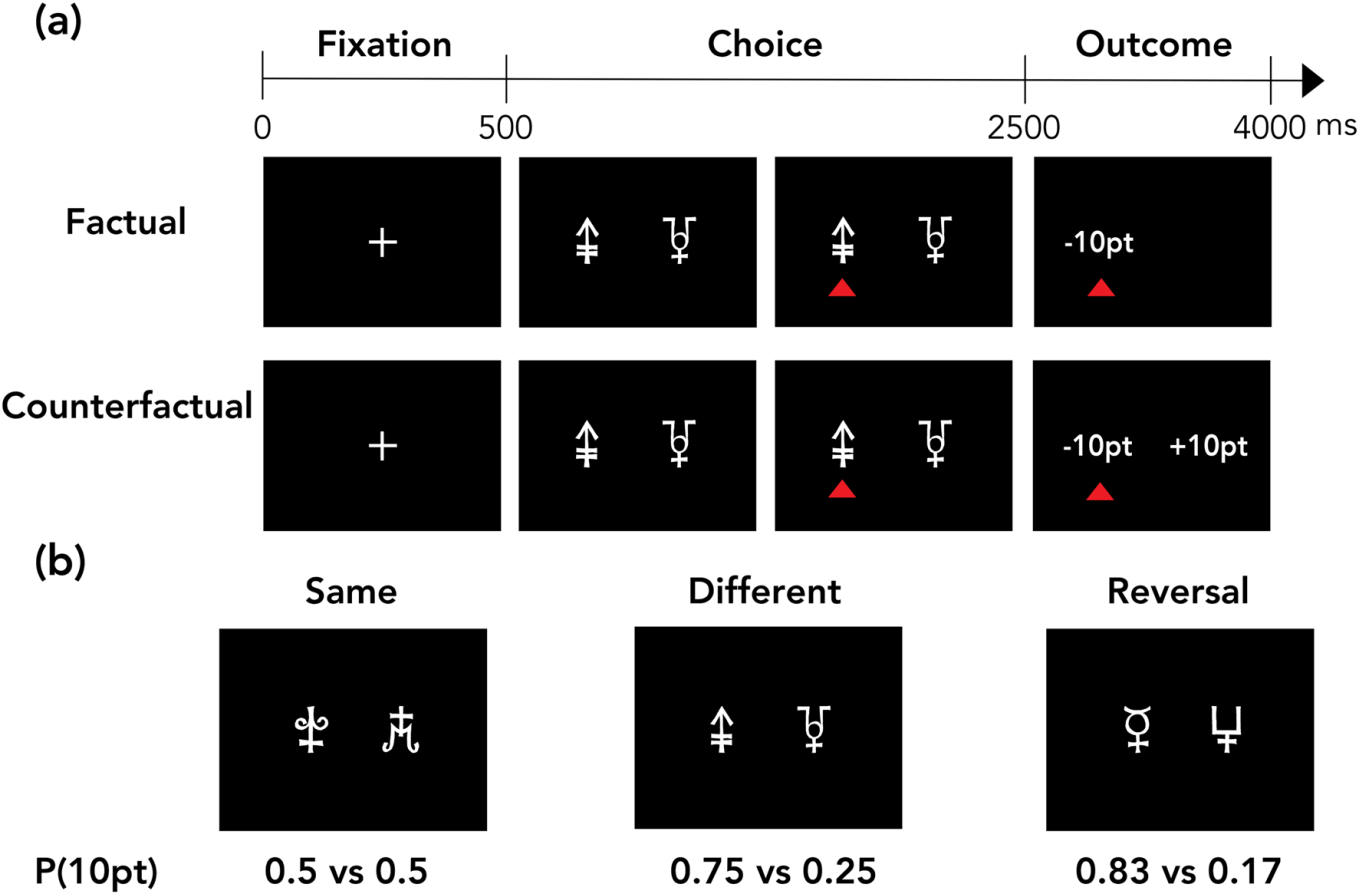
Experimental task. (**a**) There were two types of learning contexts in the present study. In the factual learning context, the subjects were shown only the outcome of the chosen option. In the counterfactual learning context, the subjects were shown the outcomes of both the chosen and unchosen options. (**b**) *Task conditions* Under the same condition, the option pair had an identical reward contingency. Under the different condition, one option had a higher reward probability than the other option. Under the reversal condition, the reward probability was reversed between the options after the first 12 trials were completed.

This study used a modified version of the probabilistic instrumental learning task developed in previous studies^10,11,24^ in both simulation and web-based experiments. First, we briefly explain the structure of this learning task (see the “Methods” section for more details). The framework used in this task is generally called a two-armed bandit problem in which an agent (subject) sequentially explores the best choice from two options^5^. This task consisted of the following two learning contexts: factual and counterfactual learning contexts (Fig. 1a). In the factual learning context, each agent was only shown the outcome of the chosen option. In the counterfactual learning context, each agent was shown the outcomes of both the chosen and unchosen options. Each agent performed two sessions in each learning context and completed 96 trials in each session. Furthermore, each session had the following three different conditions according to the different combinations of the reward probabilities of the two options: same, different, and reversal conditions. Under the same condition, both options were associated with a 50% reward probability. Under the different condition, one option was associated with a 25% reward probability, and the other option was associated with a 75% reward probability. Under the reversal condition, one option was associated with a 17% reward probability, while the other option was associated with an 83% reward probability during the first half of trials, and then, these contingencies were reversed during the remaining trials (Fig. 1b).

### Models

In this study, we mainly used three types of reinforcement learning models. All models were modifications of a typical Q-learning model. (1) The Asymmetry model has two independent learning rates, i.e., $\alpha_{c}^{ + } \;{\text{and }}\;\alpha_{c}^{ - }$, for positive and negative reward prediction errors (RPEs), respectively, to represent asymmetric value updating. (2) The Perseverance model includes the computational process of choice history independent of the outcome-based learning process. The computational process of choice history has the following two free parameters: decay rate (*τ*) and perseverance parameter (*φ*). (3) The Hybrid model has the features of both the Asymmetry and Perseverance models. In the counterfactual learning context, the models consider the impact of the forgone outcomes of the unchosen option and the impact of the obtained outcome of the chosen option. Thus, the Asymmetry and Hybrid models have two independent learning rates for each outcome (i.e., $\alpha_{c}^{ + }$, $\alpha_{c}^{ - }$, $\alpha_{u}^{ + }$, and $\alpha_{u}^{ - }$), in the counterfactual learning context. The details of these models are provided in the “Methods” section.

### Model identifiability and the usefulness of the Hybrid model

By conducting simulations, we investigated the identifiability of the three models (i.e., Asymmetry, Perseverance, and Hybrid) in each learning context, whether pseudo-asymmetric learning rates and pseudo-perseverance occurred by fitting mismatched models, and whether the Hybrid model could distinguish asymmetric value updating from choice perseveration.

To determine the identifiability of the models, we applied the three models to simulated data from the following versions of the three models: Asymmetry model assuming positivity/confirmation bias, Asymmetry model assuming negativity/opposite confirmation bias, Perseverance model, Hybrid model assuming positivity/confirmation bias, and Hybrid model assuming negativity/opposite confirmation bias. Then, we compared these models using log marginal likelihood (LML). Except for the simulated data from the Hybrid model assuming confirmation bias in the counterfactual context (rmANOVA, *F*(1.94,192.33) = 0.39, *p* = 0.67), the true model was deemed the best model (rmANOVA, *Fs* ≥ 143.27, *ps* < 6.23 × 10^–27^; see Supplementary Table S1).

**Figure 2:**
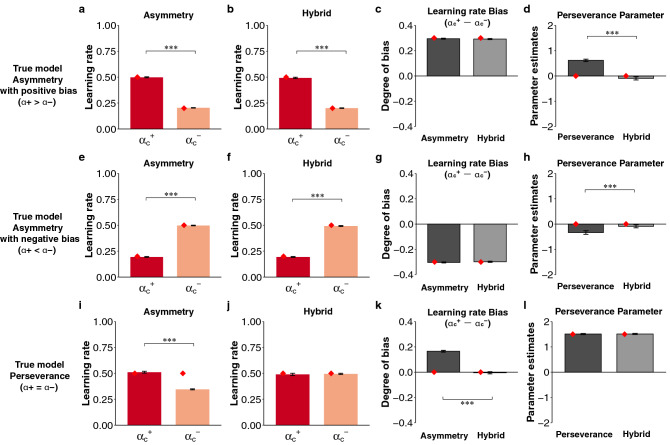
The results of the simulation in the factual learning context. (**a**–**d**) The results of the true model with asymmetric learning rates assuming positivity bias ($\alpha_{c}^{ + } = 0.5, \alpha_{c}^{ - } = 0.2$). (**e**–**h**) The results of the true model with asymmetric learning rates assuming negativity bias ($\alpha_{c}^{ + } = 0.2, \alpha_{c}^{ - } = 0.5$). (**i**–**l**) The results of the true model assuming symmetric learning rates ($\alpha_{c}^{ + } = \alpha_{c}^{ - } = 0.5$) and choice perseverance (*φ* = 1.5). (**a**,**e**,**i**) The first column indicates the learning rates in the Asymmetry model. (**b**,**f**,**j**) The second column indicates the learning rates ($\alpha_{c}^{ + }$, $\alpha_{c}^{ - }$) in the Hybrid (gradual) model. (**c**,**g**,**k**) The third column shows the degree of learning rate bias ($\alpha_{c}^{ + } - \alpha_{c}^{ - }$). (**d**,**h**,**l**) The final column shows the perseverance parameter (*φ*) in the Perseverance (gradual) and Hybrid (gradual) models. ***p < 0.001, **p < 0.01 and *p < 0.05. The error bars represent the standard error of the mean. The diamonds denote the ground-truth value of the parameters used in the data generation.

**Figure 3:**
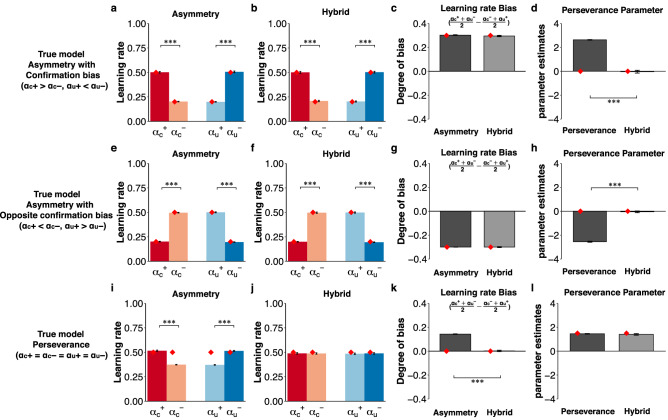
The results of the simulation in the counterfactual learning context. (**a**–**d**) The results of the true model with asymmetric learning rates assuming confirmation bias ($\alpha_{c}^{ + } = 0.5,\alpha_{c}^{ - } = 0.2, \alpha_{u}^{ + } = 0.2,\alpha_{u}^{ - } = 0.5$). (**e**–**h**) The results of the true model with asymmetric learning rates assuming opposite confirmation bias ($\alpha_{c}^{ + } = 0.2,\alpha_{c}^{ - } = 0.5, \alpha_{u}^{ + } = 0.5,\alpha_{u}^{ - } = 0.2$). (**i**–**l**) The panel shows the results of the true model with symmetric learning rates ($\alpha_{c}^{ + } = \alpha_{c}^{ - } = \alpha_{u}^{ + } = \alpha_{u}^{ - } = 0.5$) and choice perseverance (*φ* = 1.5). (**a**,**e**,**i**) The first column indicates the learning rates ($\alpha_{c}^{ + }$, $\alpha_{c}^{ - } , \alpha_{u}^{ + }$, $\alpha_{u}^{ - }$) in the Asymmetry model. (**b**,**f**,**j**) The second column indicates the learning rates in the Hybrid (gradual) model. (**c**,**g**,**k**) The third column indicates the degree of confirmation bias $\left( {\frac{{{\upalpha }_{{\text{c}}}^{ + } + {\upalpha }_{{\text{u}}}^{ - } }}{2} - \frac{{{\upalpha }_{{\text{c}}}^{ - } + {\upalpha }_{{\text{u}}}^{ + } }}{2}} \right)$. (**d**,**h**,**l**) The final column shows the perseverance parameter (*φ*) in the Perseverance (gradual) and Hybrid (gradual) models. ***p < 0.001, **p < 0.01 and *p < 0.05. The error bars represent the standard error of the mean. The diamonds denote the ground-truth value of the parameters used in the data generation.

Katahira^14^ demonstrated that by fitting the Asymmetry model to simulated data generated from the Perseverance model, the pseudo-asymmetry of the learning rates was observed. However, whether pseudo-perseverance might appear when the Perseverance model is fitted to the simulated data generated from the true Asymmetric model remains unclear. To examine this question, we fitted the Perseverance model to the simulated data from the Asymmetry model assuming positivity/confirmation bias and the Asymmetry model assuming negativity/opposite confirmation bias. In both cases, a higher perseverance parameter was observed despite the lack of perseverance (*φ* = 0) in the true model in the factual (Fig. 2d, one-sample *t*-test, *t*(99) = 12.60, *p* = 2.20 × 10^–16^; Fig. 2h, one-sample *t*-test, *t*(99) =  − 4.76, *p* = 6.75 × 10^–6^) and counterfactual (Fig. 3d, one-sample *t*-test, *t*(99) = 107.97, *p* = 1.62 × 10^–104^; Fig. 3h, one-sample *t*-test, *t*(99) =  − 58.49, *p* = 1.37 × 10^–78^) contexts. Although the Asymmetry model obviously captured true learning rate biases in the simulated data from the Asymmetry model assuming positivity/confirmation bias (Fig. 2a, paired *t*-test, *t*(99) = 56.07, *p* = 7.97 × 10^–77^; Fig. 3a, rmANOVA, *F*(1,99) = 4842.84, *p* = 6.98 × 10^–86^) and the Asymmetry model assuming negativity/opposite confirmation bias (Fig. 2e, paired *t*-test, *t*(99) =  − 52.45, *p* = 4.78 × 10^–74^; Fig. 3e, rmANOVA, *F*(1,99) = 55408.02, *p* = 6.95 × 10^–138^), we also replicated the previous finding by showing that pseudo-asymmetry of learning rates occurred when the Asymmetry model was fitted to the simulated data from the Perseverance model (Fig. 2i, paired *t*-test, *t*(99) = 16.82, *p* = 8.95 × 10^–31^; Fig. 3i, rmANOVA, *F*(1,99) = 1754.38, *p* = 8.60 × 10^–65^). These results indicate that an inadequate model causes either pseudo-asymmetric learning rates or pseudo-perseverance.

Finally, we investigated whether the Hybrid model could dissociate these underlying processes (i.e., asymmetric value updating and perseverance). Our results clearly demonstrate that the Hybrid model could capture the genuine process underlying choice behavior. When the Hybrid model was fitted to the simulated data generated from the true Asymmetry model assuming positivity/confirmation and negativity/opposite confirmation bias, the bias of learning rates was captured by the Hybrid model (Fig. 2b,c, Asymmetry vs Hybrid with paired *t*-test, *t*(99) = 1.61, *p* = 0.11; Fig. 2f,g, Asymmetry vs Hybrid with paired *t*-test, *t*(99) =  − 1.35, *p* = 0.18; Fig. 3b,c, Asymmetry vs Hybrid with paired *t*-test, *t*(99) = 1.07, *p* = 0.29; Fig. 3f,g, Asymmetry vs Hybrid with paired *t*-test, *t*(99) = 0.05, *p* = 0.96), and the pseudo-perseverance induced by fitting the Perseverance model was controlled (Fig. 2d, paired *t*-test, *t*(99) = 11.90, *p* = 8.25 × 10^–21^; Fig. 2h, paired *t*-test, *t*(99) =  − 4.50, *p* = 1.84 × 10^–5^; Fig. 3d, paired *t*-test, *t*(99) = 24.56, *p* = 6.65 × 10^–44^; Fig. 3h, paired *t*-test, *t*(99) =  − 36.16, *p* = 7.35 × 10^–59^). When the Hybrid model was fitted to the simulated data generated from the true Perseverance model, the pseudo-bias of learning rates induced by fitting the Asymmetry model was controlled (Fig. 2j,k, Asymmetry vs Hybrid with paired *t*-test, *t*(99) = 20.56, *p* = 1.69 × 10^–37^; Fig. 3j,k, Asymmetry vs Hybrid with paired *t*-test, *t*(99) = 17.88, *p* = 9.07 × 10^–33^), while the perseverance parameter was captured by the Perseverance model (Fig. 2l, paired *t*-test, *t*(99) =  − 0.49, *p* = 0.63; Fig. 3l, paired *t*-test, *t*(99) = 0.82, *p* = 0.42). Furthermore, when the Hybrid model was fitted to the simulated data generated from the true Hybrid model assuming positivity/confirmation and negativity/opposite confirmation bias, the Hybrid model identified the true parameters related to both asymmetric updating and perseverance in each learning context (see Supplementary Figs. S1, S2). These data confirm that the Asymmetry, Perseverance, and Hybrid models were identifiable. Given that the advantage of the Hybrid model was validated, we subsequently applied the empirical data collected in the web-based experiment and open data from previous studies.

### Application of the Hybrid model to empirical data

Our subsequent aim was to evaluate the extent to which asymmetric updating and choice perseverance influence actual human choice behavior. To reliably achieve this goal, we conducted a web-based experiment to obtain a relatively large sample size (*N* = 143 per context; see details in the “Methods” section). The behavioral performances measured by the correct rate and preferred response rate (see Supplementary Methods) indicated that the subjects successfully performed these tasks (see Supplementary Results and Supplementary Fig. S3).

#### Model comparisons using web-based experimental data

In addition to the three models used in the simulation (Asymmetry, Perseverance, and Hybrid models), a standard RL model was fitted to the empirical datasets as a benchmark for the model comparisons. Furthermore, we used two variants of the Perseverance and Hybrid models. The original models used in the simulation have a gradual decay rate (0 ≦ *τ* ≦ 1) in which several preceding choices influence the current choice. In addition to these Perseverance and Hybrid models with a gradual decay rate, we also fitted Perseverance and Hybrid models with an impulsive decay rate (*τ* = 1) in which only the immediately preceding choice influences the current choice because this type of decay rate was included in the models reported in a previous study^11^. Thus, we applied six models (i.e., RL, Asymmetry, Perseverance (impulsive), Hybrid (impulsive), Perseverance (gradual), and Hybrid (gradual) models) to the subjects’ choice behavior and then compared these models using log marginal likelihood (LML; see Supplementary Table S2).

In the factual learning context, the Perseverance model was the best among the six models (rmANOVA; *F*(1.73,246.04) = 17.69, *p* < 0.001; post hoc comparison: *ps* < 0.05) but was comparable with the Hybrid (gradual) model (post hoc comparison: *p* > 0.99). In the counterfactual learning context, the log marginal likelihood was decreased in the order of Perseverance (gradual), Hybrid (gradual), Asymmetry, Perseverance (impulsive), Hybrid (impulsive), and RL models (rmANOVA; *F*(1.74,246.92) = 31.09, *p* < 0.001). The Perseverance (gradual) model was the best among the six models (post hoc comparisons: *ps* < 0.05). These results indicate that the preceding choices greatly influenced the current choice in both learning contexts.

#### Parameter estimates using web-based experiment data

**Figure 4:**
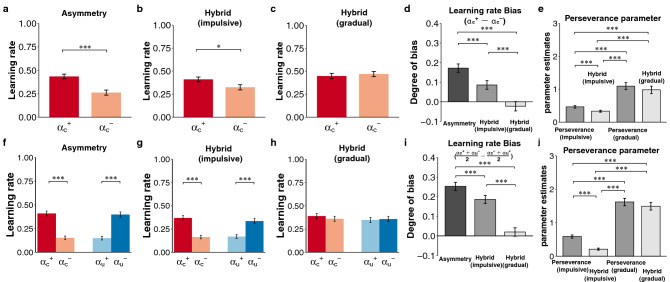
The results of the web-based experiment. The rows show the results of the web-based experiment in the factual and counterfactual learning contexts. The first to third columns represent the learning rates in the Asymmetry (**a**,**f**), Hybrid (impulsive) (**b**,**g**), and Hybrid (gradual) models (**c**,**h**). (**d**,**i**) The fourth column indicates the degree of learning rate bias (i.e., positivity bias in the factual context and confirmation bias in the counterfactual context). (**e**,**j**) The final column shows the perseverance parameter (*φ*) in the Perseverance (impulsive), Perseverance (gradual), Hybrid (impulsive), and Hybrid (gradual) models. ***p < 0.001, **p < .01 and *p < 0.05. The error bars represent the standard error of the mean.

To empirically confirm that the Hybrid model can evaluate the degree of asymmetric value updating by controlling the pseudo-bias of learning rates, we compared the estimated learning rates among the three models (Asymmetry, Hybrid (impulsive), and Hybrid (gradual) models; see Supplementary Table S3). We predicted that if the bias of learning rates estimated by fitting the Asymmetry model was pseudo-bias, this bias should disappear by fitting the Hybrid (gradual) model. This prediction was confirmed in the factual learning context. The positivity bias of learning rates ($\alpha_{c}^{ + } > \alpha_{c}^{ - }$) observed in the Asymmetry model (Fig. 4a; *t*(142) = 4.70, *p* = 6.20 × 10^–6^) disappeared by fitting the Hybrid (gradual) model (Fig. 4c; *t*(142) =  − 0.54, *p* = 0.59) but not by fitting the Hybrid (impulsive) model (Fig. 4b; *t*(142) = 2.26, *p* = 0.03). Indeed, the degree of positivity bias decreased in the order of the Asymmetry, Hybrid (impulsive), and Hybrid (gradual) models (Fig. 4d; *F*(1.42,202.94) = 37.26, *p* < 1.76 × 10^–11^; post hoc comparisons: all *ps* < 5.80 × 10^–6^). In the counterfactual learning context, our prediction was also confirmed. According to a previous study^11^, confirmation bias in RL is characterized as follows: the learning rates of the outcome that supports one’s choice (i.e., learning rate of the positive outcome of the chosen option ($\alpha_{c}^{ + }$) and negative outcome of the unchosen option ($\alpha_{u}^{ - }$) are higher than the learning rates that do not support one’s choice (i.e., learning rate of the negative outcome of the chosen option ($\alpha_{c}^{ - }$) and positive outcome of the unchosen option ($\alpha_{u}^{ + }$)). The confirmation bias of the learning rates observed in the Asymmetry model (Fig. 4f; a two-way repeated-measures ANOVA; interaction: *F*(1,142) = 155.21, *p* = 1.54 × 10^–24^) was diminished by fitting the Hybrid (gradual) model (Fig. 4h; interaction: *F*(1,142) = 0.85, *p* = 0.36) but not by fitting the Hybrid (impulsive) model (Fig. 4g; interaction: *F*(1,142) = 83.73, *p* = 5.53 × 10^–16^). The degree of confirmation bias $\left( {\frac{{{\upalpha }_{{\text{c}}}^{ + } + {\upalpha }_{{\text{u}}}^{ - } }}{2} - \frac{{{\upalpha }_{{\text{c}}}^{ - } + {\upalpha }_{{\text{u}}}^{ + } }}{2}} \right)$ was significantly decreased in the order of the Asymmetry, Hybrid (impulsive), and Hybrid (gradual) models (Fig. 4i; *F*(1.28, 182.05) = 59.70, *p* = 2.89 × 10^–15^; post hoc comparisons: all *ps* < 1.97 × 10^–8^).

Furthermore, to confirm that the Hybrid model can evaluate the degree of choice perseverance by controlling pseudo-perseverance, we examined the perseverance parameter (*φ*) in the four models (Perseverance (impulsive), Perseverance (gradual), Hybrid (impulsive), and Hybrid (gradual) models; see Supplementary Table S3). In the factual learning context, the perseverance parameters in the Perseverance (gradual) and Hybrid (gradual) models were comparable (Fig. 4e; rmANOVA: *F*(1.62,230.24) = 38.90, *p* = 3.69 × 10^–13^; post hoc comparisons: *p* = 0.12) but significantly higher than those in the Perseverance (impulsive) and Hybrid (impulsive) models (*ps* < 1.03 × 10^–6^). Similarly, in the counterfactual learning context, the perseverance parameters in the Perseverance (gradual) and Hybrid (gradual) models were comparable (Fig. 4j; rmANOVA: *F*(1.77, 251.69) = 111.47, *p* = 1.26 × 10^–32^; post hoc comparisons: *p* = 0.13) but significantly higher than those in the Perseverance (impulsive) and Hybrid (impulsive) models (*ps* < 1.37 × 10^–14^).

Taken together, these results indicate that choice perseverance mainly governed choice behavior in the web-based experiment. This result also highlights that the Hybrid model allowed us to clarify a genuine process underlying the empirical choice data.

#### Parameter recovery using web-based experimental data

While it is important to identify the perseverance parameter (*φ*) in the Perseverance (gradual) and Hybrid (gradual) models, it is possible that the perseverance parameter and inverse temperature parameter (*β*) represent a trade-off (see Eq. (4) in the “Methods” section). To determine the identifiability of these parameters, we calculated the correlation between the estimated parameters and further performed parameter recovery under both the factual and counterfactual conditions (see “Methods” section). The correlation analysis ensured that the perseverance parameter was not significantly correlated with the inverse temperature parameter in both learning contexts in both the Perseverance (gradual) and Hybrid (gradual) models (Fig. S4; *ps* > 0.99). The parameter recovery also indicated that all parameters were well recovered in the factual (Supplementary Fig. S5; 0.84 < *r* < 0.98, all *ps* < 0.001 with Bonferroni correction) and counterfactual (Supplementary Fig. S5; 0.75 < *r* < 0.95, all *ps* < 0.001 with Bonferroni correction) learning contexts. These results confirm that the parameter optimization procedure used in this study allowed us to identify the free parameters in each model.

#### Model-neutral analysis

Katahira (2018) proposed the use of a model-neutral analysis to examine the existence of the asymmetric value updating process without the RL model framework. This analysis utilizes the fact that the asymmetric learning rate induces an interaction effect between past outcomes on the current choice. The merit of a model-neutral analysis is that it does not assume a specific functional form regarding how past experience influences the reward, while RL model fitting does make this assumption. Thus, there is a possibility that the absence of the asymmetric learning rate in the RL model fitting is due to a mismatch of the functional form. To examine this possibility, we performed a model-neutral analysis (see details in Supplementary methods) of the empirical choice data. Consequently, no evidence of asymmetric value updating was observed, which is consistent with our RL model-based analysis (see detailed results in Supplementary Results).

### Application of the Hybrid model using open data

As shown above, the Hybrid model allowed us to identify a genuine process underlying the empirical choice data. Here, to reconsider the processes underlying open datasets collected by previous studies reporting asymmetric value updating, we re-analyzed these open datasets by applying the Hybrid model. Similar to the web-based experiment, we fitted six models (the RL, Asymmetry, Perseverance (impulsive), Perseverance (gradual), Hybrid (impulsive), and Hybrid (gradual) models) to these open datasets and compared the parameter estimates.

#### Dataset 1 (Palminteri et al.^11^)

Dataset 1 comprised the open data reported by Palminteri et al.^11^, who examined the asymmetric learning rates in both the factual and counterfactual learning contexts. The model comparisons (Supplementary Table S4) showed that the Perseverance (gradual) model was the best among the models in both the factual (*F*(1.11,21.16) = 6.41, *p* = 0.02) and counterfactual (*F*(1.39,26.32) = 26.58, *p* = 3.98 × 10^–6^) learning contexts.

**Figure 5:**
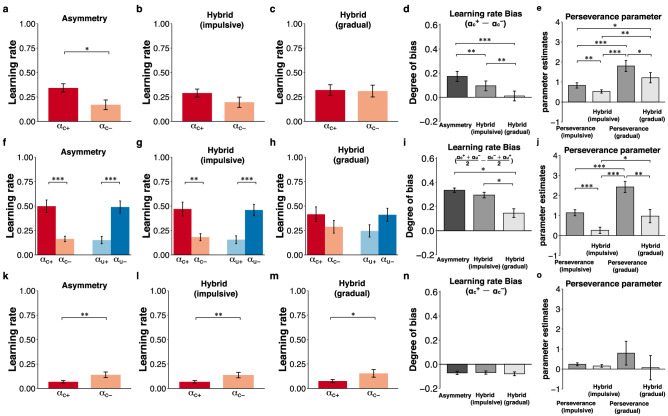
The results of open datasets 1 and 2. (**a**–**e**) The results of open dataset 1 (Palminteri et al.) in the factual learning context. (**f**–**j**) The results of open dataset 1 in the counterfactual learning context. (**k**–**o**) The results of open dataset 2 (Niv et al. 2012). The first to third columns indicate the learning rates ($\alpha_{c}^{ + }$ and $\alpha_{c}^{ - }$ in the factual learning context; $\alpha_{c}^{ + }$,$\alpha_{c}^{ - } ,\alpha_{u}^{ + }$, and $\alpha_{u}^{ - }$ in the counterfactual context) in the Asymmetry (**a**,**f**,**k**), Hybrid (impulsive) (**b**,**g**,**l**), and Hybrid (gradual) (**c**,**h**,**m**) models. (**d**,**i**,**n**) The fourth column shows the degree of learning rate bias. (**e**,**j**,**o**) The final column shows the perseverance parameter (*φ*) in the Perseverance (impulsive), Perseverance (gradual), Hybrid (impulsive), and Hybrid (gradual) models. ***p < 0.001, **p < 0.01 and *p < 0.05. The error bars represent the standard error of the mean.

In the factual learning context, by fitting the Asymmetry model, we replicated the finding showing that the learning rate of the positive outcome ($\alpha_{c}^{ + } )$ was significantly higher than that of the negative outcome $(\alpha_{c}^{ - } )$ (Fig. 5a; paired *t*-test, *t*(19) = 2.36, *p* = 0.03), supporting positivity bias. However, this positivity bias ($\alpha_{c}^{ + } - \alpha_{c}^{ - }$) was decreased by fitting the Hybrid (impulsive) model (Fig. 5b; paired *t*-test, *t*(19) = 1.35, *p* = 0.19) and was diminished by fitting the Hybrid (gradual) model (Fig. 5c; paired *t*-test, *t*(19) = 0.15, *p* = 0.88). Indeed, the degree of positivity bias was significantly smaller in the order of the Asymmetry, Hybrid (impulsive), and Hybrid (gradual) models (Fig. 5d; *F*(1.53,28.98) = 15.95, *p* = 7.52 × 10^–5^; post hoc comparisons, all *ps* < 4.47 × 10^–3^). Although the degree of the perseverance parameter significantly differed among the models (Fig. 5e; rmANOVA, *F*(2.05, 39.04) = 16.09, *p* = 6.81 × 10^–6^; post hoc comparisons, all *ps* < 0.047), the perseverance parameters (*φ*) estimated in the Perseverance and Hybrid models were above zero, leading to repeat preceding choices.

In the counterfactual learning context, we also replicated the finding showing that the learning rate of positive RPE was greater than that of negative RPE in terms of the chosen outcomes (i.e., $\alpha_{c}^{ + } > \alpha_{c}^{ - }$), but the opposite was observed in terms of the unchosen outcomes (i.e., $\alpha_{u}^{ + } < \alpha_{u}^{ - }$) (Fig. 5f; two-way rmANOVA, interaction: *F*(1,19) = 124.88, *p* = 8.54 × 10^–10^), indicating confirmation bias. Although this confirmation bias was also observed by fitting the Hybrid (impulsive) (Fig. 5g; *F*(1,19) = 53.45, *p* = 6.21 × 10^–7^) and Hybrid (gradual) models (Fig. 5h; *F*(1,19) = 5.58, *p* = 0.03), a significant difference in the learning rates was not observed between the positive and negative RPE of both the chosen and unchosen outcomes in the Hybrid (gradual) model (*ps* > 0.15). The degree of confirmation bias in the Hybrid (gradual) model was significantly smaller than that in the Hybrid (impulsive) model (Fig. 5i; rmANOVA, *F*(1.21, 23) = 7.10, *p* = 0.010; post hoc comparisons, *p* = 0.04). The perseverance parameter in the Hybrid (gradual) model was smaller than that in the Perseverance (gradual) model (Fig. 5j; rmANOVA, *F*(1.69, 32.16) = 25.98, *p* = 6.03 × 10^–7^; post hoc comparison, *p* = 1.43 × 10^–3^) but remained positive.

According to these results, the view claimed in the previous study (i.e., the existence of asymmetry in the learning rate) was not supported. In contrast, our results suggest that the choice behavior in Dataset 1 was mainly governed by choice perseverance rather than asymmetric value updating in both the factual and counterfactual learning contexts.

#### Dataset 2 (Niv et al.^8^)

Dataset 2 comprised the open data reported by Niv et al.^8^, who applied the Asymmetry model to explain risk-seeking/aversion behaviors in the factual learning context. In contrast to Dataset 1, the Asymmetry model was better than the Hybrid (impulsive) and Hybrid (gradual) models (see Supplementary Table S4; rmANOVA, *F*(1.43,21.52) = 4.29, *p* = 0.04; post hoc comparisons, *ps* < 3.01 × 10^–3^) but did not significantly differ from the RL, Perseverance (impulsive), and Perseverance (gradual) models (*ps* ≥ 0.41).

As Niv et al. reported, in the Asymmetry model, the learning rate of positive RPE ($\alpha_{c}^{ + }$) was significantly lower than that of negative RPE ($\alpha_{c}^{ - }$) (Fig. 5k; *t*(15) =  − 3.07, *p* = 7.80 × 10^–3^). This negativity bias were also observed in the Hybrid (impulsive) (Fig. 5l; *t*(15) = -3.06, *p* = 7.93 × 10^–3^) and Hybrid (gradual) models (Fig. 5m; *t*(15) =  − 2.84, *p* = 0.012). The degree of negativity bias ($\alpha_{c}^{ + } - \alpha_{c}^{ - }$) was comparable among these models (Fig. 5n; rmANOVA, *F*(1.01,15.19) = 0.75, *p* = 0.40). Additionally, the perseverance parameter (*φ*) in the Hybrid (gradual) model was almost zero and did not significantly differ from that in the Perseverance (impulsive), Perseverance (gradual), and Hybrid (impulsive) models (Fig. 5o; rmANOVA, *F*(1.55, 23.23) = 0.87, *p* = 0.41). Thus, our results based on the Hybrid model support the asymmetric value updating process claimed in a previous study (Niv et al.^8^).

## Discussion

This study considered a method to dissociate two factors underlying human choice behavior, i.e., asymmetric learning and choice perseverance. By using these methods, we attempted to identify the processes underlying human choice behavior. In the simulation, we replicated previous findings^14^ showing that pseudo-asymmetric updating was induced when a model without perseverance (Asymmetry model) was fit to simulated data from a model with symmetric updating and perseverance (Perseverance (gradual) model). In contrast, when a model without perseverance was fitted to the simulated data generated from a model with true asymmetric updating, pseudo-perseverance was observed. As Katahira^14^ mentioned, these results show that asymmetric updating and choice perseverance result in similar choice behavior statistical properties. Therefore, it is important to investigate how to dissociate these processes underlying choice behavior. In this study, we considered the Hybrid model, which incorporating both asymmetric updating and perseverance components, and we tested the capability of the Hybrid model using simulated and empirical datasets. The simulations showed that the Hybrid model could identify the following true parameters in the simulated dataset generated from all hypothetical models: optimistic asymmetric updating, pessimistic asymmetric updating, and symmetric updating with perseverance. The Hybrid model also identified the true parameters of the simulated dataset from a hypothetical model containing asymmetric updating and perseverance. These results support the advantage of the Hybrid model in distinguishing the processes underlying choice behavior.

Palminteri et al.^11^ claimed that asymmetric value updating underlies choice behavior in a probabilistic instrumental learning task. Their candidate models also included the Perseverance model and showed that an asymmetric learning rate model attained a better fit than the Perseverance model^12^. However, their Perseverance model only considered impulsive perseverance (the influence of only the most recent choice under the same condition). As Katahira^14^ noted, a model that considers only impulsive perseverance is insufficient for avoiding statistical bias in estimates of the learning rate. Thus, there is a possibility that the overlooked influence of a more distant past induces pseudo-asymmetric learning rates.

To determine whether learning asymmetry or perseverance is dominant in choice behavior in a probabilistic instrumental learning task while addressing the above issue, we applied the Hybrid model (with gradual perseverance) to the empirical data. To obtain the empirical data, we mainly focused on data collected in a web-based experiment involving relatively large samples (*N* = 143 per context; compared with the previous study, *N* = 20 per context) to improve the statistical robustness. As previously reported^10,11^, we replicated the asymmetry in learning rates in both factual and counterfactual learning contexts in a model without the perseverance factor (Asymmetry model). The learning rates of the chosen outcomes when the outcomes were positive were greater than those when the outcomes were negative, whereas the opposite pattern was observed in the learning rates of the unchosen outcomes. Such asymmetry was interpreted as “confirmation bias” in a previous study^11^. However, we found that this asymmetry in learning rates disappeared when the Hybrid model was fitted, including the gradual perseverance factor (*τ* < 1) and the asymmetric learning rate (Hybrid (gradual) models. Moreover, the model-neutral analysis did not support the existence of asymmetric value updating. These findings support our previous claim that model misspecification in which perseverance is not considered in the model can cause the erroneous detection of asymmetry in the learning rates of choice behavior^14^. Our results also highlight the merit of the Hybrid model in identifying the underlying process in empirical data.

We also showed that when the Hybrid model, which included impulsive perseverance (*τ* = 1), was fitted, the asymmetric learning rates were significant in both contexts. Furthermore, we demonstrate that this residual asymmetry of learning rates disappeared when using the Hybrid model that incorporated gradual perseverance (*τ* < 1). Indeed, similar results were obtained using open data in a previous study^11^. These findings suggest that the superiority of the asymmetric learning model over the perseverance model in the previous study was due to an insufficient length of the choice history.

Furthermore, we demonstrated that the Hybrid model could identify asymmetric updating in empirical data obtained in a different type of task. In the open data reported by Niv et al.^8^, the asymmetry in the learning rates remained after controlling for choice perseverance. The factor inducing asymmetry in value updating in the context of reinforcement learning remains unclear. It is possible that the structural differences in the instrumental learning tasks might contribute to the discrepancy between the two datasets of open data in the influence of choice perseverance. In Niv et al.^8^, the existence of forced choices might have weakened the effect of choice history^23^. Furthermore, the existence of certain options that vary the risk level between the options might lead to asymmetric value updating. Future studies should investigate the psychological source of asymmetric learning rates.

Throughout this study, we used the frequentist statistical tests to compare the results with the previous studies collecting open datasets used in this study. Recent methodological studies alerted that the statistical strategy which takes a two-step approach, such as estimating individual model parameters and then performing statistical analyses using frequentist methods, could produce further statistical biases^25^. Nevertheless, our simulations clearly showed the usefulness of the Hybrid model, because this model could capture the true model parameters without estimation bias.

In conclusion, we demonstrate the utility of the Hybrid model with multiple computational components in dissociating the cognitive process underlying human choice behavior. The proposed model used in this study contributes to a deeper understanding of the neural mechanisms of and individual differences in these cognitive components in instrumental learning.

## Methods

### Behavioral tasks

In this study, we used the same behavioral task in both the simulations and web-based experiment. The task was a modified version of the probabilistic instrumental learning task developed in previous studies^10,11,24^. The framework used in this task is generally called a two-armed bandit problem in which an agent (subject) sequentially explores the best choice among multiple options^5^. This task consisted of a factual block and a counterfactual block (Fig. 1a). In the web-based experiment, half of the subjects started with the factual block, and the other half started with the counterfactual block. In each block, the agent experienced two sessions separated by a 20-s break. In each session, we selected eight abstract stimuli (Agathodaimon font) and generated four different pairs. In the second session, all stimuli were renewed such that the agent began learning anew (i.e., the option stimuli differed between the two sessions). The display positions of the stimuli were set to appear on the left and right in the same number of trials. These four stimulus pairs were distributed among the following three conditions: same (1 pair), different (2 pairs), and reversal (1 pair). Under the same condition, both stimuli were associated with a 50% reward probability (here, the reward was “+ 10 pt”). Under the different condition, one stimulus was associated with a 25% reward probability, and the other stimulus was associated with a 75% reward probability. Under the reversal condition, one stimulus was associated with a 17% reward probability, and the other stimulus was associated with an 83% reward probability during the first 12 trials, and then, these contingencies were reversed during the final 12 trials (Fig. 1b). Each pair was presented in 24 trials per session. Thus, each session included 96 trials. The order of the trials was pseudo-randomized with the constraint that the same condition continued for no more than four times in a sequence. The agents were not given any explicit information regarding the reward probabilities. The agents were instructed to earn as many points as possible across experiments by trial and error.

The agents completed an 8-trial practice session before each block (factual or counterfactual learning) after the overall task description was provided. The stimuli used in the practice trials were not used in the main task. At the initiation of each trial, a fixation crosshair appeared for 500 ms. Following the fixation crosshair, one of four stimulus pairs was displayed for 2000 ms during which the agent had to choose one of the two stimuli by pressing either “F” (left option) or “J” (right option) on their keyboard. If the agent chose one option within 1500 ms, a red triangle was placed below the chosen option until the outcome presentation. If the agent did not choose any option within 1500 ms, a warning message was displayed for 1500 ms, and the trial was considered missed (“− 10 pt”). Then, the outcomes were displayed for 1500 ms (“+ 10 pt” or “− 10 pt”). In the factual learning context, the agents were only shown the outcome of the chosen option. In the counterfactual learning context, the agents were shown the outcomes of both the chosen and unchosen options. Since this research involved subjects in a web-based experiment, some tasks reported in Palminteri et al.^11^ were modified. The main modifications were the inclusion of a time limit for the response and the use of a fixed duration for the feedback presentation. In previous experiments, the subjects responded and observed feedback at their own pace. These modifications aimed to control the entire duration of the experiment.

### Models

We fit several models, including the asymmetric learning rate and choice perseverance. For the data from the factual learning task, we used the following six RL models: standard RL, Asymmetry, Perseverance (impulsive), Perseverance (gradual), Hybrid (impulsive), and Hybrid (gradual) models. The details of the models are described below.

The standard RL model is the most basic of all models considered in the present study. In the standard RL model, the action value of the chosen option in trial $t$, which is denoted by $Q_{c} \left( t \right)$, is updated according to the following equation:

1 $$ Q_{c} \left( {t + 1} \right) = Q_{c} \left( t \right) + \alpha (R_{c} \left( t \right) - Q_{c} \left( t \right)). $$

Here, the outcome (of the chosen option) in trial $t$ is denoted by $R_{c} \left( t \right)$. $(R_{c} \left( t \right) {-}{ }Q_{c} \left( t \right))$ represents the prediction error, which is subsequently denoted by $\delta_{c}$. The learning rate (*α*) determines how much the model updates the action value with the prediction error. The initial action value of each option is set to zero. For the data from the factual learning task, only the Q value of the chosen option is updated because the agents are informed only of the outcome of the chosen option. Choice probability $P_{{\text{c}}} \left( t \right)$ is determined by the following softmax function:

2 $$ P_{{\text{c}}} \left( t \right){ } = { }\frac{1}{{1 + {\text{exp}}\left( { - \beta \left( {Q_{c} \left( t \right) - Q_{u} \left( t \right)} \right)} \right)}}, $$

where $Q_{c}$ is the Q value of the chosen option, and $Q_{u}$ is the value of the unchosen option. The inverse temperature (*β*) determines the sensitivity of the choice probabilities to the difference between the Q values.

The Asymmetry model is extended from the standard RL model to allow the learning rates to differ ($\alpha_{c}^{ + }$, $\alpha_{c}^{ - }$) depending on the sign of the prediction error. Thus, the Q values are updated as follows:

3 $$ Q_{c} \left( {t + 1} \right) = { }\left\{ {\begin{array}{*{20}c} {Q_{c} \left( t \right) + \alpha_{c}^{ + } \delta_{c} \left( t \right) if \delta_{c} \left( t \right) \ge 0} \\ {Q_{c} \left( t \right) + \alpha_{c}^{ - } \delta_{c} \left( t \right) if \delta_{c} \left( t \right) < 0} \\ \end{array} } \right.. $$

Previous studies have shown that this model can be used to express positivity bias or confirmation bias^10,11^.

The Perseverance model uses the same update rule as the standard RL model (Eq. (3)). In the models that incorporate the perseverance factor, the choice trace $C\left( t \right)$ is defined to introduce the effect of a past choice to the choice probability^19,26^:

4 $$ P_{{\text{c}}} \left( t \right){ } = { }\frac{1}{{1 + \exp \left( { - \beta \left( {Q_{c} \left( t \right) - Q_{u} \left( t \right)} \right) - \varphi (C_{c} \left( t \right) - C_{u} \left( t \right)} \right))}}. $$

The perseverance parameter (*φ*) is a parameter that controls for the tendency to repeat the choice of or avoid a recently chosen option. A high positive value of this parameter indicates that the agent frequently repeats the previous choice. The choice trace is computed using the following update rule^14,21^:

5 $$ \begin{array}{*{20}c} {C_{c} \left( {t + 1} \right) = C_{c} \left( t \right) + \tau \left( {1 - C_{c} \left( t \right)} \right)} \\ {C_{u} \left( {t + 1} \right) = C_{u} \left( t \right) + \tau \left( {0 - C_{u} \left( t \right)} \right)} \\ \end{array} , $$

where $C_{c}$ and $C_{u}$ denote the choice trace of the chosen and unchosen options, respectively. The decay rate determines the number of preceding choices in the choice history influencing the current choice^14,27^. In the Perseverance (impulsive) model, with the decay rate fixed at 1, only the immediately preceding choice influences the current choice. Most previous studies examining choice perseverance have incorporated the influence of only the immediate prior trial^20,28^. However, Katahira^14^ showed that the long-term choice history caused bias in the estimation of the asymmetric learning rates.

The Hybrid model is a model combining the Asymmetry and Perseverance models. This model incorporates not only the asymmetric learning rates but also the choice trace.

For the data from the counterfactual learning task, we used the following six RL models as described in the factual learning task: the standard RL, Asymmetry, Perseverance (impulsive), Perseverance (gradual), Hybrid (impulsive), and Hybrid (gradual) models. Here, all models are allowed to update the Q values of both the chosen and unchosen options because the agent was informed of both outcomes. The standard RL, Perseverance (gradual), and Perseverance (impulsive) models have the same parameters as the models used in the factual learning task because an identical learning rate is used to update the values of both the chosen and unchosen options regardless of the sign of the prediction error.

In the Asymmetry models, four different learning rates are defined to represent the asymmetric updating of the chosen ($\alpha_{c}^{ + }$, $\alpha_{c}^{ - }$) and unchosen ($\alpha_{u}^{ + }$, $\alpha_{u}^{ - }$) options. The Q values of the chosen and unchosen options are computed as follows:

For the chosen option (see Eq. (3)).

For the unchosen option:

6 $$ Q_{u} \left( {t + 1} \right) = { }\left\{ {\begin{array}{*{20}c} {Q_{u} \left( t \right) + \alpha_{u}^{ + } \delta_{u} \left( t \right) if \delta_{u} \left( t \right) \ge 0} \\ {Q_{u} \left( t \right) + \alpha_{u}^{ - } \delta_{u} \left( t \right) if \delta_{u} \left( t \right) < 0} \\ \end{array} } \right., $$

where $\delta_{u}$ denotes the prediction error of the unchosen option.

In the counterfactual learning context, we also used the Hybrid (gradual) and Hybrid (impulsive) models, which are Hybrid models combining the Asymmetry model and the Perseverance model, to examine the asymmetry of the learning rate while incorporating choice perseverance.

### Parameter estimation and model comparison

Using the R function “solnp” in the Rsolnp package^29^, we fit the parameters of each model with the maximum a posteriori (MAP) estimation and calculated the log marginal likelihood of each model using Laplace approximation^30^. In contrast to a likelihood, a marginal likelihood penalizes a complex model with extra parameters in the marginalization process. Because the marginal likelihood is proportional to the posterior probability of the model, the model resulting in the highest marginal likelihood is the most likely one given a particular data set. Notably, this situation is only true if all models have an equal prior probability (i.e., all models are equally likely before the data are provided). This method incorporates the prior distributions of the parameters and can avoid extreme values in the estimates of the parameters^31,32^. The prior distributions and constraints were set following Palminteri et al.^11^. All learning rates were constrained to the range of 0 ≤ $\alpha$ ≤ 1 with a *Beta* (1.1, 1.1) prior distribution. The inverse temperature was constrained to the range of $\beta$ ≥ 0 with a *Gamma* (shape = 1.2, scale = 5.0) distribution. In the perseverance model, the decay rate was constrained to the range of 0 ≤ $\tau$ ≤ 1 with a *Beta* (1, 1) distribution (i.e., a uniform distribution), and the perseverance parameter was constrained to the range of − 10 ≤ $\varphi$ ≤ 10 with a *Norm* (*μ* = 0, *σ*^2^ = 5) distribution.

### Simulations

To understand how the Hybrid model works, we conducted simulations that directly evaluated the amount of bias in the parameter estimates of the misspecified models. In the simulations, we first generated the choice data under the five simulated conditions (true models; see Supplementary Table S1) used to perform the probabilistic instrumental learning task (see the ‘Behavioral tasks’ section) and then fitted three models (the Asymmetry, Perseverance (gradual), and Hybrid (gradual) models) to the data.

In the factual learning context, the simulated conditions from the versions of the three models were set as follows: (i) a model with asymmetric learning rates assuming positivity bias ($\alpha_{c}^{ + } = 0.5$, $\alpha_{c}^{ - } = 0.2$,$\beta = 0.3$, $\tau = 0.4$, $\varphi = 0$); (ii) a model with an asymmetric learning rate assuming negativity bias ($\alpha_{c}^{ + } = 0.2$, $\alpha_{c}^{ - } = 0.5$, $\beta = 0.3$, $\tau = 0.4$, $\varphi = 0$); (iii) a model with a symmetric learning rate and perseverance ($\alpha_{c}^{ + } = \alpha_{c}^{ - } = 0.5$, $\beta = 0.3$, $\tau = 0.4$, $\varphi = 1.5$); (iv) a model with an asymmetric learning rate and perseveration assuming positivity bias ($\alpha_{c}^{ + } = 0.5$, $\alpha_{c}^{ - } = 0.2$, $\beta = 0.3$, $\tau = 0.4$, $\varphi = 1.5$); and (v) a model with an asymmetric learning rate and perseveration assuming negativity bias ($\alpha_{c}^{ + } = 0.2$, $\alpha_{c}^{ - } = 0.5$, $\beta = 0.3$, $\tau = 0.4$, $\varphi = 1.5$). In the counterfactual learning context, the simulated conditions from the versions of the three models were set as follows: (i) a model with an asymmetric learning rate assuming confirmation bias ($\alpha_{c}^{ + } = 0.5$, $\alpha_{c}^{ - } = 0.2$, $\alpha_{u}^{ + } = 0.2$, $\alpha_{u}^{ - } = 0.5$, $\beta = 0.3$, $\tau = 0.4$, $\varphi = 0$); (ii) a model with an asymmetric learning rate assuming opposite confirmation bias ($\alpha_{c}^{ + } = 0.2$, $\alpha_{c}^{ - } = 0.5$, $\alpha_{u}^{ + } = 0.5$, $\alpha_{u}^{ - } = 0.2$, $\beta = 0.3$, $\tau = 0.4$, $\varphi = 0$); (iii) a model with a symmetric learning rate and perseverance ($\alpha_{c}^{ + } = \alpha_{c}^{ - } = \alpha_{u}^{ + } = \alpha_{u}^{ - } = 0.5$, $\beta = 0.3$, $\tau = 0.4$, $\varphi = 1.5$); (iv) a model with an asymmetric learning rate and perseveration assuming confirmation bias ($\alpha_{c}^{ + } = 0.5$, $\alpha_{c}^{ - } = 0.2$, $\alpha_{u}^{ + } = 0.2$, $\alpha_{u}^{ - } = 0.5$, $\beta = 0.3$, $\tau = 0.4$, $\varphi = 1.5$); and (v) a model with an asymmetric learning rate and perseveration assuming opposite confirmation bias ($\alpha_{c}^{ + } = 0.2$, $\alpha_{c}^{ - } = 0.5$, $\alpha_{u}^{ + } = 0.5$, $\alpha_{u}^{ - } = 0.2$, $\beta = 0.3$, $\tau = 0.4$, $\varphi = 1.5$). All parameters were set according to the parameters obtained from the web-based experiment. The number of trials was set as 960 trials per session per block. Under each simulation condition, 100 virtual datasets were simulated.

### Web-based experimental procedures

One hundred and fifty adults participated in the web-based experiment via CrowdWorks (https://crowdworks.jp/). We limited the subjects’ age to over 18 years and paid approximately 700 yen (approximately $6) if the subjects completed all tasks and surveys without any interruption. Informed consent was obtained from all subjects by clicking ‘I Agree’ after reading the information regarding the aim and procedures of this study. After the subjects provided their basic demographic information, including gender and age, and downloaded Inquisit player (Millisecond Software LLC, Seattle, USA), the subjects started the behavioral task (see the ‘Behavioral tasks’ section). The subjects were anonymized, and their privacy was protected. The study was approved by the Ethical Research Committee of Nagoya University, and the study was carried out in accordance with the relevant guidelines and regulations.

Seven subjects were excluded due to inappropriate task execution. Six of these subjects showed a false start rate greater than 30%. Thus, these subjects pressed any button before the choice options were presented in more than 30% of the trials. The other subject chose only the option that appeared on the right side across the experiments (even though each option randomly appeared on both sides). Thus, data from 143 subjects (58 females and 85 males) aged between 19 and 72 years (mean ± SD = 38.7 ± 9.6) were included in the subsequent analyses.

### Parameter correlation and parameter recovery

To validate our model-fitting results in the web-based experiment, we checked the correlations between the free parameters in each learning context and the capacity of recovering the model parameters using simulated data^33,34^. For the parameter recovery, we simulated the choice dataset under each condition of our behavioral paradigm with model parameters corresponding to those estimated from our actual subjects (*N* = 143). The number of trials was set as 960 trials per session per block. These simulations were conducted using the model parameters estimated using the Asymmetry, Perseverance (gradual), and Hybrid (gradual) models. Thus, 143 virtual datasets were simulated per context and model. We fitted the same model used in the simulation to the simulated datasets. Then, the correlation coefficients between the true parameters used in the simulation and the estimated parameters in each context and model were calculated. Additionally, to determine the precision of the parameter recovery, we calculated the root mean squared error between the true value used to generate the data and the estimated value.

### Additional open data analysis

To clarify the genuine process underlying the empirical choice data collected in previous studies reporting asymmetric updating, we also applied the Hybrid model to two open datasets. Dataset 1 comprised the open data reported by Palminteri et al.^11^. Since our research was carried out according to this previous study, the experimental procedures were mostly the same. Although the authors of the previous study^11^ analyzed the influence of choice perseverance, they did not examine the influence of the gradual perseverance factor (*τ* < 1). Thus, using these open data, tests were performed by comparing the models, including those incorporating the gradual perseverance factor. Furthermore, we used Dataset 2 reported by Niv et al.^8^ to investigate whether the asymmetric learning rates observed in another learning task could be explained by choice perseverance. We also applied the Hybrid model to these previous data and compared the model fitting and learning rate parameters. More detailed information regarding each dataset is as follows.

#### Dataset1 (Palminteri et al.^11^; https://figshare.com/authors//2803402)

In Palminteri et al.^11^, the asymmetric learning rates were examined in both factual and counterfactual learning contexts. As mentioned above, our web-based study was carried out using largely the same procedures as those used in this previous study. However, in the previous task, the subjects responded and observed feedback at their own pace. Furthermore, the previous study employed a between-subjects design in which each subject performed the task in either a factual (*N* = 20) or counterfactual (*N* = 20) learning context.

#### Dataset2 (Niv et al.^8^: http://www.princeton.edu/~nivlab/data/NivEtAl2012JNeuro/)

In Niv et al.^8^, Asymmetry models were used to explain risk-seeking/aversion behaviors in a factual learning context. A negative outcome learning rate higher than a positive outcome learning rate leads to risk aversion, whereas the opposite pattern leads to risk seeking. Their task included the following six option pairs that differed in risk and expected rewards: 20¢ (100%) versus 0 (50%) /40¢ (50%), 40¢ (100%) versus 0 (50%) /40¢ (50%), 20¢ (100%) versus 40¢ (100%), 0¢ (100%) versus 0 (50%) /40¢ (50%), 0¢ (100%) versus 20¢ (100%), and 0¢ (100%) versus 0¢ (100%). The experiment involved two types of trials. In the ‘choice trials,’ the subjects were required to choose between two stimuli, whereas in the ‘forced trials,’ the subjects were presented only one of five stimuli and had to choose the presented stimulus (*N* = 16). Similar to the analyses of the web-based data, we compared the estimated parameters among the Asymmetry, Hybrid (impulsive), and Hybrid (gradual) models.

### Statistical tests

For the model comparison, one-way repeated-measures analysis of variance (rmANOVA) was conducted to compare the log marginal likelihoods of the models in each learning context (factual and counterfactual learning). We also investigated the difference in learning rates ($\alpha_{c}^{ + }$, $\alpha_{c}^{ - }$) within each model. For the Asymmetry and Hybrid models in the factual learning context, the difference in the learning rates was compared by a paired t-test. For the Asymmetry and Hybrid models in the counterfactual learning context, two-way rmANOVAs with Valence (positive or negative) and Choice (chosen or unchosen) were performed to test for differences in the four learning rates ($\alpha_{c}^{ + }$, $\alpha_{c}^{ - }$, $\alpha_{u}^{ + }$, and $\alpha_{u}^{ - }$). The degree of biases in the learning rates were compared across the models by using a one-way rmANOVA in each learning context. Additionally, the degree of the perseverance parameter (*φ*) was compared across the models using a one-way rmANOVA in each learning context. To correct for the violation of the sphericity assumption, Greenhouse-Geiser’s adjustment of the degrees of freedom was used in all rmANOVAs when appropriate. The post hoc pairwise comparisons were performed based on Shaffer’s correction for multiple comparisons. For the simulation, the differences between the true and estimated parameters were evaluated by using a one-sample *t*-test with the true parameters. To control for the multiple comparison issue, the significance of the one-sample *t*-tests was tested with Bonferroni correction. In the parameter correlation analysis, we estimated the Pearson’s correlation coefficients between the model parameters of the Perseverance (gradual) and Hybrid (gradual) models in the factual and counterfactual learning contexts. Additionally, in the parameter recovery, we estimated the Pearson’s correlation coefficients between the model parameters estimated from the empirical dataset and the simulated dataset. The significance of the correlation coefficients was tested with Bonferroni correction to avoid multiple comparison issues. These analyses were executed using R version 3.5.1 statistical software (http://cran.us.r-project.org).

## Supplementary Information

Supplementary Information.

## Data Availability

The data supporting the findings of this study are available in figshare at https://figshare.com/articles/Cognitive_bias_and_perseverance/10042319.
