# Supplementary material for: Dissociation between asymmetric value updating and perseverance in human reinforcement learning
Source: Sci Rep. 2021 Feb 11;11:3574. doi: 10.1038/s41598-020-80593-7 (PMC7878894; doi:10.1038/s41598-020-80593-7)
Supplement: Supplementary file 1 — Supplementary Information. [file 41598_2020_80593_MOESM1_ESM.docx]

**Supplementary information**

**Dissociation between asymmetric value updating and perseverance in human reinforcement learning**

**Michiyo Sugawara and Kentaro Katahira**

Kentaro Katahira

E-mail: katahira.kentaro@b.mbox.nagoya-u.ac.jp (KK)

**This PDF file includes the following:**

Supplementary Figures 1 to 5

Supplementary Tables 1 to 6

**Supplementary methods**

## *Performance evaluation in the web-based experiment*

To evaluate whether the subjects successfully performed the behavioral tasks, we calculated the preferred response rate under the same condition and the correct rate under the different and reversal conditions as described in a previous study^1^. The preferred response rate was calculated as the fraction of “preferred response,” which was defined as the most frequently chosen option (i.e., the option chosen by the subject in more than 50% of the trials). The correct rate was defined as the fraction of trials in which the subjects chose the option associated with a higher reward probability.

We divided each session (24 trials) into four phases (6 trials per phase) to investigate the learning-related performance changes under each condition. Because the subjects completed two sessions in each learning context, we pooled the trials in each phase of the two sessions (i.e., 12 trials per pooled phase). Then, the correct rate under the different and reversal conditions and the preferred response rate under the same condition were calculated in each pooled phase.

## *Model-neutral analysis of the web-based experiment*

To assess the asymmetric value updating process underlying the empirical choice data collected in the web-based experiment, we conducted a model-neutral analysis as proposed in Katahira (2018)^2^. Because a detailed explanation was provided in the previous study^2^, here, we only briefly introduce the concept of this analysis. As reported in previous studies^2,3^, one behavioral consequence of asymmetric learning rates is that the impact of past outcomes depends on subsequent outcomes. Thus, an interaction likely exists between the outcome of one trial ago and the outcome of two trials ago that serves as a factor influencing the current choice. In this case, since the regression model assumes that the outcomes of one and two trials ago affect the current choice independently of each other, this point can be used to test the existence of learning rate asymmetry in the data. Thus, the regression coefficient of the interaction term is 0 if the true learning rate is symmetric ($\alpha^{+}{=\alpha}^{-}$). Here, we only consider up to two trials ago and consider the condition under which the outcome is a binary value (1 = reward, 0 = no reward). Under this condition, the interaction term represents the degree of the asymmetric learning rates. A negative interaction indicates positive asymmetric updating ($\alpha^{+}{>\alpha}^{-}$), while a positive interaction indicates negative asymmetric updating ($\alpha^{+}{<\alpha}^{-}$).

To create the data used in this analysis, we first sorted the trial sequences of each subject, each four-stimulus pair, and each session. From each sequence, we extracted all possible three successive trials (we refer to these as a ‘triplet’), denoted by ($t+1$)-th trial, $t$-th trial, and ($t-1$)-th trial. Then, from the resulting triplets, we further selected only the triplets in which the same option was selected at both the $t$-th trial and $(t-1)$-th trial. Finally, the triplets containing time out (no response) trials were excluded, and the remaining triplets were included in the analysis. Then, we constructed a logistic regression model to predict the probability that a subject chooses the same option in the $(t+1$)-th trial as in the $t$-th trial, denoted by $p(stay\left( t+1 \right))$. In the factual learning condition, the regressors are the outcomes of the choice at the $t$-th trial ($Rc\left( t \right)$) and ($t-1)$-th trial ($Rc\left( t-1 \right)$) and their interaction term$Rc\left( t \right)\times Rc\left( t-1 \right)$. The regressors are coded as $Rc\left( t \right)=1: rewarded and Rc\left( t \right)=0$: unrewarded. The logistic regression model is as follows:

**Equation (1)**

$$\log\frac{p(stay\left( t+1 \right))}{p(switch\left( t+1 \right))} = b_{0}+b_{1}Rc\left( t \right)+b_{2}Rc\left( t-1 \right)+b_{12}Rc\left( t \right)Rc\left( t-1 \right)$$

where $p(switch\left( t+1 \right))$ is the probability that the subject switches the choice at the ($t+1$)-th trial. The intercept $b_{0}$ represents the overall tendency to repeat the same choice, which may absorb the effects of the choices and rewards at trials before the ($t-1)$-th trial. In the counterfactual learning context, the logistic regression model also includes the regressors of the outcomes of the unchosen options at the $t$-th trial ($Ru\left( t \right)$) and the ($t-1$)-th trial ($Ru\left( t-1 \right)$) and their interaction ($Ru\left( t \right)\times Ru\left( t-1 \right)$). Thus, the logistic regression model is expressed as follows:

**Equation (2)**

$$\log\frac{p(stay\left( t+1 \right))}{p(switch\left( t+1 \right))}=b_{0}+b_{1}Rc\left( t \right)+b_{2}Rc\left( t-1 \right)+b_{3}Ru\left( t \right)+b_{4}Ru\left( t-1 \right)$$

$$+b_{12}Rc\left( t \right)Rc\left( t-1 \right)+b_{34}Ru\left( t \right)Ru\left( t-1 \right)$$

The hypothesis test based on the null hypothesis b_12_ = 0 and/or b_34_ = 0 was conducted by using mixed-effects models (“glmer” function) implemented with the lme4 package^4^ in the R programming language. Within-subject factors (intercept, main effects, and their interaction) were included as random effects, i.e., allowed to vary across subjects.

**Supplementary result**s

## *Behavioral performance in the web-based experiment*

To determine whether the subjects in the web-based experiment adequately learned the probabilistic instrumental learning task, we conducted a two-way repeated-measures analysis of variance (rmANOVAs) with the effects of Context (factual and counterfactual) and Phase (1st to 4th) of the preferred response rate under the same condition and the correct rate under the different and reversal conditions (see Supplementary Figure S3).

Under the same condition, a significant main effect of Phase was observed (*F*(2.82,400) = 3.74, *p* = .01), whereas the main effect of Context and the interaction were not significant (Context: *F*(1,142) = 2.21, *p* = .14; Context × Phase: *F*(2.71,385.31) = 3.74, *p* = .71). The post hoc comparisons showed that the preferred rate significantly increased from the 1st to 3rd phase (*p* < .01). Under the different condition, the main effect of Phase and the interaction were significant (Phase: *F*(2.76,392.35) = 30.07, *p* < .001; Context × Phase: *F*(2.69,382.62) = 4.50, *p* < .01), and no main effect of Context was observed (*F*(1,142) = 2.68, *p* = .10). Significant simple effects of Phase were observed in both the factual and counterfactual contexts (factual: *F*(2.71,384.91) = 9.82, *p* < .001; counterfactual: *F*(2.63,373.86) = 28.26, *p* < .001). Additionally, significant simple effects of Context were found in the 2nd and 3rd phases (2nd: *F*(1,142) = 6.79, *p* < .05; 3rd: *F*(1,142) = 7.25, *p* < .001). The post hoc comparisons showed that the correct rate significantly increased from the 1st to 2nd (*p* < .05) and from the 3rd to 4th (*p* <.05) phases in the factual learning context, whereas the correct rate increased only from the 3rd to 4th phases (*p* <.05) in the counterfactual learning context. These results suggest that the subjects successfully learned the reward probabilities of the presented options. Under the reversal condition, the main effect of Phase was significant (*F*(2.16,306.59) = 102.5, *p* < .001), but the main effect of Context and the interaction were not (Context: *F*(1,142) = .002, *p* = .97; Context × Phase: *F*(2.3,326.03) = 1.56, *p* = .21). The post hoc comparisons indicated that the correct rate significantly increased in the 2nd phase, decreased in the 3rd phase, and then increased again in the 4th phase (*ps* < .001). This profile confirmed that the subjects detected the reversal in the reward probability.

## *Model-neutral analysis of the web-based experiment*

In the factual learning context, the logistic regression model included the following three terms: the outcome of the chosen option at the $t$-th trial (***Rc_t_***), the chosen outcome at the ($t-1$)-th trial (***Rc_t-1_***), and the interaction between these past outcomes (***Rc_t_***×***Rc_t-1_***). The regression coefficients of ***Rc_t_*** and ***Rc_t-1_*** were significant and positive (***Rc_t_***: *β* = .89, *p* < .001; ***Rc_t-1_***: *β* = .40, *p* < .001, Table S6). However, the interaction was not significant (***Rc_t_***×***Rc_t-1_***: *β* = -.08, *p* = .30), indicating that evidence of asymmetry in value updating during the underlying learning process was lacking. In addition, the intercept was significant and positive (*β* = .27, *p* < .001), suggesting a tendency to repeat choices.

In the counterfactual context, the logistic regression model including the following six terms: the chosen outcome at the $t$-th trial (***Rc_t_***), the chosen outcome at the ($t-1$)-th trial (***Rc_t-1_***), the interaction between these past outcomes (***Rc_t_***×***Rc_t-1_***), the outcome of the unchosen option at the $t$-th trial (***Ru_t_***), the unchosen outcome at the ($t-1$)-th trial (***Ru_t-1_***), and the interaction between these latter two outcomes (***Ru_t_***×***Ru_t-1_***). The regression coefficients of ***Rc_t_*** and ***Rc_t-1_*** were significant and positive (***Rc_t_***: *β* = .59, *p* < .001; ***Rc_t-1_***: *β* = .27, *p* < .001, Table S6). However, the regression coefficients of ***Ru_t_*** and ***Ru_t-1_*** were significant but negative (***Ru_t_***: *β* = -.40, *p* < .001; Rut-1: *β* = -.19, *p* < .001). Furthermore, neither interactions (between the outcomes of the chosen options or between those of the unchosen options) were significant (***Rc_t_***×***Rc_t-1_***: *β* = -.011, *p* = .19; ***Ru_t_***×***Ru_t-1_***: *β* = -.09, *p* = .27), further indicating that evidence of asymmetric value updating is lacking. The intercept was significant and positive (*β* = .98, *p* < .001).

**References**

1. Palminteri, S., Lefebvre, G., Kilford, E. J. & Blakemore, S.-J. Confirmation bias in human reinforcement learning: Evidence from counterfactual feedback processing. *PLOS Computational Biology* **13**, e1005684 (2017).

2. Katahira, K. The statistical structures of reinforcement learning with asymmetric value updates. *Journal of Mathematical Psychology* **87**, 31–45 (2018).

3. Katahira, K. The relation between reinforcement learning parameters and the influence of reinforcement history on choice behavior. *Journal of Mathematical Psychology* **66**, 59–69 (2015).

4. Bates, D. *et al.* *Linear Mixed-Effects Models using ‘Eigen’ and S4*. (2019).

**Supplementary Figure S1. The results of the simulations generated from the Hybrid model (gradual) in the factual learning context.** (a - d) The results of the true model with asymmetric learning rates assuming positivity bias ($\alpha_{c}^{+}=0.5, \alpha_{c}^{-}=0.2$) and choice perseverance. (e - h) The results of the true model with asymmetric learning rates assuming negativity bias ($\alpha_{c}^{+}=0.2, \alpha_{c}^{-}=0.5$) and choice perseverance. The first and second columns indicate the learning rates ($\alpha_{c}^{+}$and $\alpha_{c}^{-}$) in the Asymmetry (a, e) and Hybrid (gradual) (b, f) models, respectively. (c, g) The third column shows the degree of learning rate bias ($\alpha_{c}^{+}- \alpha_{c}^{-}$). (d, h) The final column shows the perseverance parameter (*φ*) in the Perseverance (gradual) and Hybrid (gradual) models. ****p* < .001, ***p* < .01 and **p* < .05. The error bars represent the standard error of the mean. The diamonds denote the ground-truth value of the parameters used in the data generation.

**Supplementary Figure S2. The results of the simulations generated from the Hybrid (gradual) model in the counterfactual learning context.** (a - d) The results of the true model with asymmetric learning rates assuming confirmation bias ($\alpha_{c}^{+}=0.5,\alpha_{c}^{-}=0.2, \alpha_{u}^{+}={0.2,\alpha}_{u}^{-}=0.5$) and choice perseverance. (e - h) The results of the true model with asymmetric learning rates assuming opposite confirmation bias ($\alpha_{c}^{+}=0.2,\alpha_{c}^{-}=0.5, \alpha_{u}^{+}={0.5,\alpha}_{u}^{-}=0.2$) and choice perseverance. The first and second columns indicate the learning rates ($\alpha_{c}^{+}, \alpha_{c}^{-}, \alpha_{u}^{+}$, and $\alpha_{u}^{-}$) in the Asymmetry (a, e) and Hybrid (gradual) (b, f) models, respectively. (c, g) The third column shows the degree of learning rate bias ($\frac{\alpha_{c}^{+}+\alpha_{u}^{-}}{2}-\frac{\alpha_{c}^{-}+\alpha_{u}^{+}}{2}$). (d, h) The final column shows the perseverance parameter (*φ*) in the Perseverance (gradual) and Hybrid (gradual) models. ****p* < .001, ***p* < .01 and **p* < .05. The error bars represent the standard error of the mean. The diamonds denote the ground-truth value of the parameters used in the data generation.

**Supplementary Figure S3. Behavioral performances across the three conditions in the factual and counterfactual contexts of the web-based experiment.** We divided each learning session into four subphases. Each curve shows the average performances of 143 participants in the four phases. The error bars represent the standard errors of the mean. (a) Under the same condition, performance was measured as the preferred rate. Under both the different (b) and reversal (c) conditions, performance was measured as the correct rate. Blue and green denote the factual and counterfactual learning contexts, respectively. ****p* < .001, ***p* < .01, and **p* < .05.

**Supplementary Figure S4. Parameter correlation in the web-based experiment.** Correlation matrices indicating Pearson’s correlations between the estimated parameters. The top row was estimated from the Asymmetry model in the factual (a) and counterfactual (b) learning contexts. The bottom row was estimated from the Hybrid (gradual) model in the factual (c) and counterfactual (d) learning contexts. The color and value in each cell represent Pearson’s correlation coefficients.

**Supplementary Figure S5. Parameter recovery in the web-based experiment.** The correlation matrix represents the Pearson’s correlation coefficients between the parameters estimated from the empirical data (x-axis) and the simulated data (y-axis). (a - c) The results of the Asymmetry, Perseverance (gradual), and Hybrid (gradual) models in the factual learning context. (d - f) The results of the Asymmetry, Perseverance (gradual), and Hybrid (gradual) models in the counterfactual learning context. The color and value in each cell represent the Pearson’s correlation coefficients. Additionally, in the diagonal elements, the root mean squared errors between the true value used in the data generation and the estimated value are noted in the parentheses.

**Supplementary tables**

**Supplementary Table S1. List of models and model selection results of the simulation data**

**Supplementary Table S2. List of models and model selection results of the web-based experiment data**

**Supplementary Table S3. List of models and parameter results of the web-based experiment data**

**Supplementary Table S4. Models and model selection results of Dataset 1 (Palminteri et al., 2017) and Dataset 2 (Niv et al., 2012)**

**Supplementary Table S5.** **List of models and parameter results of Dataset 1 (Palminteri et al., 2017) and Dataset 2 (Niv et al., 2012)**

**Supplementary Table S6.** **Regression coefficients of the logistic regression model in the model-neutral analysis**
